# A Novel Background Modeling Algorithm for Hyperspectral Ground-Based Surveillance and Through-Foliage Detection

**DOI:** 10.3390/s22207720

**Published:** 2022-10-11

**Authors:** David Schreiber, Andreas Opitz

**Affiliations:** AIT Austrian Institute of Technology GmbH, 1210 Vienna, Austria

**Keywords:** border surveillance, hyperspectral sensor, background modeling, through-foliage penetration, fragmented occlusions

## Abstract

Foliage penetration is an unsolved important part of border surveillance of remote areas between regular border crossing points. Detecting penetrating objects (e.g., persons and cars) through dense foliage in various climate conditions using visual sensors is prone to high fault rates. Through-foliage scenarios contain an unprecedented amount of occlusion—in fact, they often contain fragmented occlusion (for example, looking through the branches of a tree). Current state-of-the-art detectors based on deep learning perform inadequately under moderate-to-heavy fragmented occlusion. The FOLDOUT project builds a system that combines various sensors and technologies to tackle this problem. Consequently, a hyperspectral sensor was investigated due to its extended spectral bandwidth, beyond the range of typical RGB sensors, where vegetation exhibits pronounced reflectance. Due to the poor performance of deep learning approaches in through-foliage scenarios, a novel background modeling-based detection approach was developed, dedicated to the characteristics of the hyperspectral sensor, namely strong correlations between adjacent spectral bands and high redundancy. The algorithm is based on local dimensional reduction, where the principal subspace of each pixel is maintained and adapted individually over time. The successful application of the proposed algorithm is demonstrated in a through-foliage scenario comprised of heavy fragmented occlusion and a highly dynamical background, where state-of-the-art deep learning detectors perform poorly.

## 1. Introduction

This work presents a novel background modeling (background subtraction) algorithm for a hyperspectral camera intended for ground-based outdoor surveillance. The algorithm was developed as part of the FOLDOUT (through-foliage detection, including in the outermost regions of the EU) project [1]. Surveillance of remote areas between regular border crossing points is one of the main tasks of border guards operating at the EU/Schengen’s external borders. However, the available technologies have clear limitations when it comes to surveying areas such as forests or areas with dense vegetation, which provide easy hiding places for potential underlying irregular activities [2]. In particular, through-foliage detection is an unsolved, but important part of border surveillance. Detection of objects (e.g., persons and cars) through dense foliage in various climate conditions using visual sensors is prone to high failure rates. Due to the dense foliage, FOLDOUT scenarios contain an unprecedented amount of occlusion—in fact, they often present fragmented occlusions (for example, those looking through the branches of a tree) [3]. Fragmented occlusions are much more challenging than ordinary partial occlusions, as typically only small fragments of the person are seen through the foliage. Currently, state-of-the-art person detectors based on deep learning are trained with datasets containing only partial occlusions and not fragmented occlusion. In cases of heavy fragmented occlusion, only small, scattered parts of a person are visible, which makes it impossible even for a human observer to determine a bounding box which circumscribes the location of a person appearing in the video frame. Research performed within FOLDOUT further indicates that even when augmenting the training of deep-learning-based person detectors with data containing fragmented occlusions, the resulting improvement in detecting persons is achieved only in cases of slight fragmented occlusion, but not for moderate-to-heavy fragmented occlusions [4]. Consequently, interest within FOLDOUT was also directed towards other types of detectors, e.g., motion-based methods such as background modeling. 

The motivation to employ a hyperspectral sensor for outdoor surveillance, and in particular through-foliage detection in FOLDOUT, is due to its extended bandwidth relevant for vegetation detection. As discussed in [5], vegetation shows a higher reflectance at around 540 nm compared to the ranges from 400 to 500 nm and from 640 to 660 nm, where absorption by chlorophyll takes place. It also shows a pronounced increase in reflectance around 700 nm, which lies beyond the typical range of an RGB sensor. There is only a limited amount of previous literature for both the usage of hyperspectral sensors in ground-based surveillance scenarios as well as for the application of background modeling algorithms to hyperspectral surveillance video streams. In [6], a multi-sensor approach is adapted for detecting moving targets in indoor and outdoor scenarios. However, the background modeling algorithm is employed on thermal and short-wave Infrared (SWIR) imagery, while the hyperspectral sensor is employed afterwards to classify the detected objects. In [7], the common Mixture-of-Gaussians background modeling approach is employed separately on each hyperspectral band, and a decision is obtained via consensus approach. In [8], the background estimation is based on the average of 50 frames and is computed based on a reduced representation, namely the first principal components of PCA. However, the dimensional reduction is global (i.e., for a whole dataset and where the unit data vectors are entire video frames) and the subspace dimensionality is fixed. In contrast, we employ an incremental PCA individually for each pixel. Thus, each pixel has its own principal subspace, where the subspace dimensionality varies adaptively over time. Additionally, the proposed algorithm incorporates a means to identify dynamic background pixels resulting from swaying vegetation and illumination changes. 

As mentioned above, current state-of-the-art deep-learning-based detectors perform poorly in cases of moderate-to-heavy fragmented occlusions [4]. However, for the case of hyperspectral imagery, an additional challenge exists, which is related to the accurate transformation from radiance (combination of material properties and the light source characteristics) to reflectance (material properties of the object) in uncontrolled environments. This was confirmed in additional work within FOLDOUT [5], where a trained classifier was evaluated on hyperspectral frames based on hyperspectral radiance. As calibration boards cannot be used in outdoor surveillance scenarios to learn the characteristics of the light source, one would need to either use active illumination or develop advanced methods to estimate the light source characteristics. The conclusion in [5] was that the hyperspectral deep-learning-based classifier has currently limited applicability to outdoor scenarios. This motivated the development of a novel background-based detection algorithm dedicated to hyperspectral sensors even further. Background modeling algorithms are typically pixel based, and the appearance of each background pixel is adapted over time. Therefore, changes in illumination and corresponding changes of the spectral signature do not pose a problem.

Evaluation of a challenging outdoor surveillance scenario with severe fragmented occlusion where persons are walking behind trees indicates that the novel background-based hyperspectral algorithm can detect frames depicting persons with an accuracy of 93% at a false alarm rate of 7%. Since there was no trained deep neural network (DNN)-based detector available for the hyperspectral camera, an indirect comparison was performed with DNN-based detectors applied to RGB and thermal cameras observing the same scenario at the same time. The RGB and thermal detectors were able to detect frames with persons with an accuracy of 12% and 3%, respectively (with a false alarms rate of 0%). 

There have been advances in the field of hyperspectral sensor technology since the purchase of the XISPEC-KIT-SM5X5-NIR hyperspectral sensor within FOLDOUT in 2019. It is likely that hyperspectral sensors with reduced noise and channels intercorrelation, increased resolution and reduced costs will be used more commonly in the future in ground-based surveillance. 

Our contribution is twofold: (i) we introduce a novel dedicated background modeling algorithm for a hyperspectral camera, and (ii), we apply the hyperspectral sensor and the novel algorithm to ground-based surveillance scenarios of FOLDOUT, namely, through-foliage detection on border crossings, which contain a large amount of fragmented occlusion. There have hardly been any previous attempts to develop a background modeling algorithm suited for a hyperspectral camera, nor to use this sensor in ground-based surveillance, not to mention the challenging though-foliage scenarios characterized by heavy fragmented occlusions. 

The rest of this paper is organized as follows. In Section 2, related work concerning background modeling is outlined. In Section 3, first the passive hyperspectral sensor is described (Section 3.1), followed by details of its noise measurements (Section 3.2). Section 3.3 outlines methods of dimensional reduction investigated for the hyperspectral sensor, including batch-wise (Section 3.3.1) and incremental (Section 3.3.2) methods. Section 3.4 evaluates the subspace dimensionality of our hyperspectral sensor, while Section 3.5 describes the novel background modeling algorithm. In particular, Section 3.5.1 outlines the motivation and the state-of-the-art regarding background modeling for visual sensors, while Section 3.5.2 provides a detailed description of the algorithm and its parametrization. Section 4 describes the experiment performed. Finally, Section 5 presents the experimental evaluation, while Section 6 contains conclusions and future work. 

## 2. Related Works

*Background modeling* (or *background subtraction*) and the related foreground detection (or change detection) are important steps in video surveillance and well-established themes in computer vision (for an exhaustive survey, see the book [9]). Conventional background modeling methods exploit the temporal variation of each pixel to model the background. Due to the dynamic nature of real-world scenes, noise and illumination variations, Gaussian Mixture Models became a popular solution [10]. However, as the distribution of pixel values over time cannot be modeled accurately by a mixture of Gaussians, non-parametric models that estimate background probability density functions at individual pixel locations were introduced [11]. To reduce memory requirements of the kernel density estimation involved, the observations were clustered into codewords [12]. Alternatively, the sample consensus method was used to store a short sample history for each pixel [13]. The use of a local binary descriptor and its variants to improve the spatial awareness has also been studied [14]. Regarding the use of feedback mechanisms to adjust parameters on the fly, many parametric methods use local variance analysis to guide segmentation behavior and to trigger model update mechanisms. An exception, for example, is the work of [15], which restrains parameter adjustments to regions with unstable segmentation behavior. However, they use ad hoc formulas and parametrization for the update models. 

For a recent survey regarding deep neural networks in the context of background modeling, see [16]. Deep neural networks have been demonstrated to be a powerful framework for background subtraction in video acquired by static cameras. However, deep neural networks require high computational overhead, special hardware such as expensive GPUs and extensive datasets for training. Moreover, deep neural networks outperform previous background modeling methods when they are trained with a scene-specific dataset [17]. These limitations render deep neural networks-based background modeling methods impractical for border surveillance applications such as the target of the FOLDOUT project. 

There is little previous literature concerning background modeling for multispectral sensors, and even less so for hyperspectral sensors. In [18], a Gaussian background modeling based on the Mahalanobis distance and spectral distance metric, as well as combining the results from several spectral bands (6–7 bands) via pooling. [19] tackle background modeling of multispectral sensors by applying mixture of Gaussians per channel. In [20], the traditional codebook approach of [12] to background modeling is extended to for background subtraction, multispectral case (up to seven bands). These approaches indicate that the hyperspectral background modeling performs favorably compared to RGB. In a multi-sensor setup [6], the background estimation is performed on thermal and SWIR sensors, and the hyperspectral sensor is employed afterwards to classify the detected regions of interest. The approach in [7] presents a hyperspectral background modeling by using a Mixture of Gaussians for each spectral band separately and obtaining a decision based on the consensus approach. In [8], the background subtraction is performed based on a reduced representation, namely over the first principal components of PCA; however, the dimensional reduction is offline and global (for a whole dataset), and the dimensionality is fixed. In contrast, we use an incremental PCA where the data elements are pixels, not whole frames, and where each pixel has its own principal subspace, and the dimensionality varies adaptively over time in accordance with the varying background of the pixel. 

Since hyperspectral data have strong correlations between adjacent spectral bands and high redundancy, dimensionality reduction techniques are required. Some previous computer vision approaches have employed subspace learning techniques for background modeling, but in an entirely different way than in this work [9]. Subspace learning typically uses a dimensional reduction technique which is applied on a collection of images to construct a background model, which is represented by the mean image. In this way, foreground segmentation is accomplished by computing the difference between the input image and its reconstruction on a subspace. In this approach, a whole frame is considered as a single data unit (typically as a 1D vector). There are many variants to this basic idea; however, each version suffers from some limitations, including: (1) It is applied to the whole frame without considering the contribution of different image parts in building the background model; (2) the application of this model is mostly limited to grayscale images, and the integration of multi-channel data is not straightforward; (3) the representation is not multi-modal, so dynamical background and illumination changes cannot be handled correctly. In contrast, over time, our approach builds and updates a background subspace for each pixel individually, thus overcoming the limitations mentioned above. 

## 3. Materials and Methods

### 3.1. The Hyperspectral Sensor 

The hyperspectral camera chosen for FOLDOUT was the XIMEA model XISPEC-KIT-SM5X5-NIR, a compact lightweight passive hyperspectral sensor, based on standard CMOS area sensors, whose interference filters are organized in a 5 × 5 pattern (25 spectral bands). The full width at half maximum of spectral bands ranges between about 5 and 20 nm. The XIMEA MQ022HG-IM-SM5X5-NIR (Generation 1; XIMEA GmbH, Münster, Germany) hyperspectral camera with a short-pass filter was selected as it featured the best tradeoff in coverage of the visible and NIR bands. 

The motivation to make use of a hyperspectral camera stems from FOLDOUT’s preoccupation with through-foliage detection. Vegetation shows a higher reflectance at around 540 nm compared to the ranges from 400 to 500 nm and from 640 to 660 nm, where absorption by the chlorophyll takes place. It also shows a pronounced increase in reflectance around 700 nm. Throughout this paper, the term *vegetation* or *foliage* refers to all plants or plant material captured by the camera. This includes leaves, branches, stems, etc., ranging from grass to bushes and trees [5]. To avoid double peaks, i.e., crosstalk between wavelength bands, an additionally provided filter limits the spectral range to the range 600–875 nm. However, as the peaks produced by the interference filters have considerable overlaps, a calibration procedure was provided by the manufacturer: a correction matrix of size 24 × 25, yielding 24 virtual bands. However, as the noise level rises and varies more considerably between the virtual spectral images than in the original ones, the original bands were used in the following.

**Figure 1:**
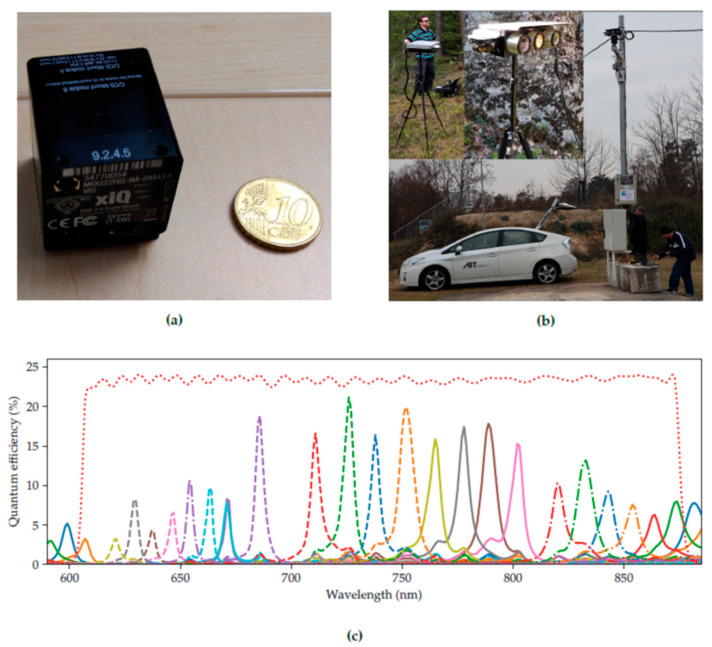
The camera system. (**a**) XIMEA MQ022HG-IM-SM5X5-NIR camera compared to a EUR 0.10 coin. (**b**) Camera system on a tripod and on a mast (top left). (**c**) Response curves of the camera (The red dotted line is the combined transfer function of the optical filters). Taken from [5].

**Figure 2:**
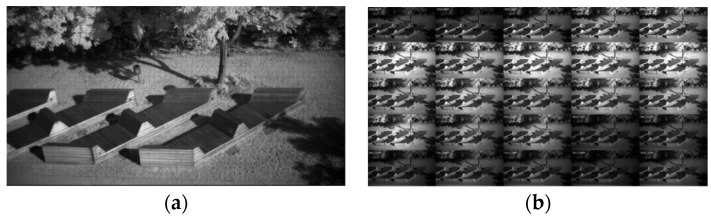
(**a**) Hyperspectral input frame and (**b**) planar hyperspectral cube after demosaicing. Taken from [5].

Figure 1 (taken from [5]) shows the compact casing of the MQ022HG-IM (Generation 1; XIMEA GmbH, Münster, Germany) series compared to a EUR 0.10 coin (Figure 1a). The hyperspectral camera was mounted in a custom-made multi-purpose multi-camera housing (Figure 1b). The housing also contained a Forward Looking Infrared (FLIR) Boson thermal camera and a FLIR Blackfly 4K RGB camera. The frames were recorded from all cameras semi-synchronously. The response curves for each band of the XIMEA MQ022HG-IM-SM5X5-NIR, as well as the filter transfer function indicating the selected wavelength range, are shown in Figure 1c. The concept of the IMEC technology is that on top of a CMOS image sensor, filters are deposited and patterned directly, organized in a 5 × 5 mosaic pattern. Each array captures the spectral information of the incident light. After a raw frame has been captured, it needs to be demosaiced to receive a hyperspectral cube; see Figure 2 (taken from [5]). The 1085 × 2045 mosaic image is decomposed into 25 spectral images (bands), each of size 217 × 409 pixels.

### 3.2. Noise Measurements

We have used the state-of-the-art paper concerning modeling noise of a CMOS camera [21]. Data processing was carried out in MATLAB. The total variance of the noise (measured in gray levels) of the sensor at each pixel $\left(i,j\right)$ is given by the sum of the variance of the noise component $\sigma_{I}^{2}\left(i,j\right)$ that depends on the signal level $I\left(i,j\right)$ and the variance of the constant component in case of a closed iris (dark current noise) $\sigma_{C}^{2}\left(i,j\right)$, namely

(1) $$\sigma_{N}^{2}\left(i,j\right)=\sigma_{I}^{2}\left(i,j\right)+\sigma_{C}^{2},$$

The variance of the constant component depends on the variance of the zero mean amplifier noise and on the quantization step of the analog-to-digital converter. The noise component that does depend on the signal level $I\left(i,j\right)$ depends on the combined gain of the camera amplifier and circuitry, the scaling (response) factor, and the number of collected electrons due to the dark current. In practice, the dark current noise is measured from images captured with a closed iris for a specific camera gain value. Given two dark current frames (captured with a closed shutter and with the same gain), the statistics of the dark current noise can be obtained from the difference image.

The dark current as well as the dark current noise $\sigma_{k}$ were computed for both the original 25 bands and for the corrected 24 virtual bands. The mean dark current and its noise are expected to be independent of the camera gain, and this indeed was observed for our sensor. Therefore, we show the mean dark current and its noise only for the gain = 0 case. To reduce overlap and crosstalk between the spectral bands, the original 25 bands can be corrected by the correction matrix provided with the camera. The correction matrix converts the 25 original bands into 24 virtual bands. However, this transformation alters the original bands in such a way, that the corrected virtual bands are no longer restricted to the range $\left[0,\;255\right]$ and contain large positive as well as large negative values. Moreover, the noise level of the virtual bands is amplified non-uniformly across the virtual bands. 

**Figure 3:**
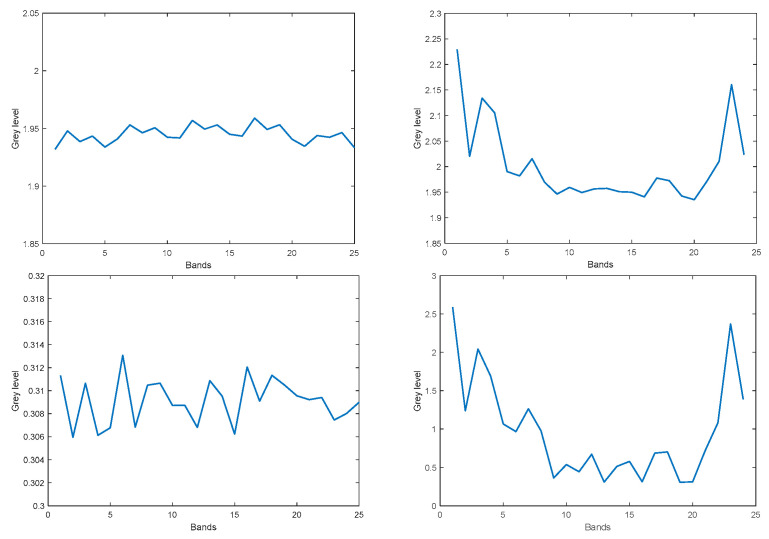
Dark current: Mean dark current for the original 25 bands (**top left**) and 24 virtual bands (**top right**). Dark noise variance for the original 25 bands (**bottom left**) and 24 virtual bands (**bottom right**).

The mean dark current for the original bands as well as for the virtual bands is fairly constant across bands and is about 1.9 gray levels. However, while the noise variance for the original bands is roughly constant across bands and is about 0.3 gray levels, the noise variance for the corrected bands varies between 0.5 and 3.5 gray levels, and is largest at the spectrum’s endpoints, as shown in Figure 3.

**Figure 4:**
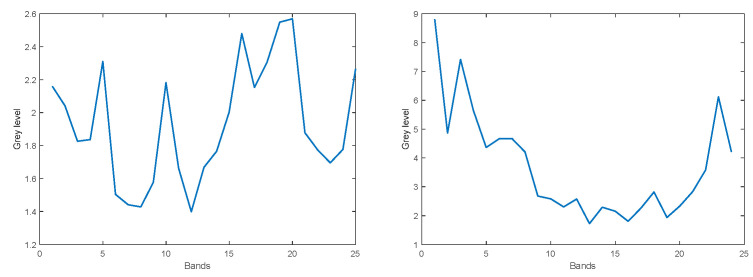
Variance of sensor noise which depends on the gray level, after vignetting correction, average gray level = 124. **Left**: for the 25 original bands. **Right**: the 24 virtual bands.

To measure the noise which depends on the gray level, a polytetrafluoroethylene (PTFE) calibration target provided with the camera was employed. To achieve an even illumination across the target, the measurements were taken outdoors. Even so, the strong vignetting observed in this sensor had to be compensated for by proper normalization per pixel. The noise which depends on the gray level was computed from the difference of two images taken at the same gray level value. The dependence of the total noise on the average gray level (or exposure) turned out to be quite linear, in accordance with [21]. Hence, we next present a comparison of the noise of the original bands and the virtual bands for a specific gray level. Figure 4 shows the average noise (variance) which depends on the gray level per image band, for an exposure of 345 microseconds, where after vignetting correction, all image bands have a constant average gray value of 124 (left: original 25 bands; right: 24 virtual bands). The noise significantly increases when attempting to correct the bands using the correction matrix provided with the camera, resulting in 24 virtual bands, and reaches unacceptable levels at the lower and upper ends of the spectrum. Consequently, it was decided to use the 25 original bands rather than the 24 virtual bands, where the overlap of the original response peaks of the original bands is to be taken care of by the dimensional reduction algorithm, described in Section 3.3.

### 3.3. Dimensional Reduction of the Hyperspectral Data

Since hyperspectral data have strong correlations between adjacent spectral bands and high redundancy information contained in hyperspectral data, even with a few tens of narrow bands, either data transformations or feature extraction including band selection or other data dimensionality reduction techniques are suggested before the hyperspectral data are used. The commonly applied transforms within the context of remote sensing are the principal components analysis (PCA) [22] and signal-to-noise ratio-based maximum noise fraction (MNF) transform [23]. Similarly, automated learning of low-dimensional linear or multi-linear models from training data has become a standard paradigm in computer vision. A variety of linear learning models and techniques such as the above-mentioned PCA, Iterative Robust Least Squares estimation (IRLS) [24], Autoregressive analysis (AR), and Singular Value Decomposition (SVD), have been widely used for the representation of high dimensional data such as appearance, shape, motion, temporal dynamics, etc. [22]. These approaches differ in their noise assumptions, the use of prior information, and the underlying statistical models, but all of them are directly or indirectly related to linear or bilinear regression. One drawback of such learning methods is that they are based on least squares estimation techniques, and hence fail to account for outliers, which are common in realistic training sets. Therefore, it is common to use an intra-sample outlier process to account for pixel outliers, namely employing robust statistics.

There are two dimensional-reduction paradigms. Batch learning refers to algorithms that use a batch of training data at once, learning the subspace offline. Incremental learning refers to algorithms that apply to streaming data collected over time, updating the learned subspace parameters for each new incoming datum. As, for our background modeling algorithm, both types (batch and incremental) of dimensional reduction are needed, we outline and compare each of them.

#### 3.3.1. Batch-Wise Dimensional Reduction Algorithms

We have tested the following batch-wise dimensional reduction methods on the hyperspectral data (as well as on synthetic data): Principal Component Analysis (PCA) [22] (original formulation as well as the robust PCA variant, using a robust estimator to remove data outliers), Maximum Noise Fraction (MNF) [23], and the robust Iterative Least Squares estimation (IRLS) [24]. These three algorithms differ in their assumptions regarding the noise distribution. The PCA assumes the same noise magnitude for all vectors and components. The MNF method assumes that the magnitude of noise differs between the components of the data and the noise magnitude of a specific component is the same for all vectors. In contrast, the IRLS aims so solve the more general case, where the noise magnitude also varies between the vectors. Our experiments indicate that the MNF shows no apparent benefit over PCA, while the IRLS turned out to be sensitive to the parametrization and initial conditions, suffers from instabilities, runs much longer, and is not significantly better than the PCA. Moreover, given that PCA produces an orthogonal basis for the principal subspace, and given that the required fast eigenvalue decomposition function (commonly using SVD) is widely available in many programming languages/frameworks), we have chosen to use PCA as our dimensional reduction method.

#### 3.3.2. Incremental Dimensional Reduction Algorithms

An incremental dimensional reduction is also required for the proposed hyperspectral background modeling algorithm (Section 3.5.2). Incremental PCA was developed within the computer vision literature. There are several versions, which differ in some mathematical details regarding how the subspace is obtained. Here, we follow the approach of [25]. Let the dimension of each data vector be denoted by *d*, and the current dimension of the principal subspace be *k*, where *k < d*. The key idea here is that at each incremental step, the entire *d* × *d* covariance matrix, and the eigen-decomposition required, is approximated by a $\left(k+1\;\right)\cdot \left(k+1\;\right)\;$covariance matrix composed of the current principal subspace and the new coming data vector, properly normalized, so as to control the relative influence of the new coming data vector and the existing principal vectors. The result of the incremental step is a new, *k +* 1 dimensional subspace. However, the eigenvector(s) corresponding to the least significant eigenvalue(s) can be omitted, depending on the reconstruction accuracy required.

### 3.4. What Is the Subspace Dimensionality of the Hyperspectral Pixels

**Figure 5:**
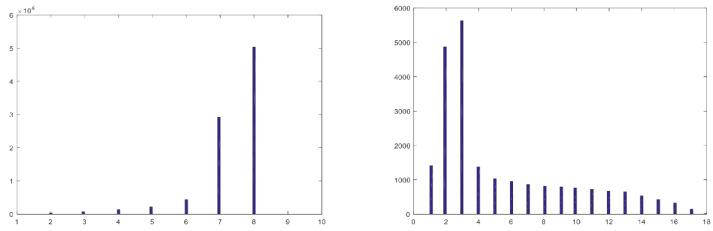
Distribution of subspace dimensionality required to achieve 98% reconstruction accuracy. **Left**: for individual pixels. **Right**: for 2 × 2 patches.

How much can the dimensionality of the hyperspectral pixels be reduced, while keeping most of the information they contain within a desired accuracy (e.g., 98%)? We distinguish between global and local dimensional reduction, namely, finding one subspace dimension that is appropriate for the temporal history of all the pixels of the frame, vs. finding the subspace dimension for each pixel’s history individually. For the global approach, we took 10 successive frames. The resulting PCA-based reconstruction achieves 98% accuracy with subspace dimensionality of five. Next, we tested local dimensional reduction. For each pixel, we performed a PCA decomposition within a moving window of width of 10 frames, along 100 consecutive frames. Figure 5 (left) shows the distribution of subspace dimensionality obtained from all the individual pixels along the 100 frames, needed to achieve 98% reconstruction accuracy for each pixel. As can be seen, a maximal subspace dimensionality of nine is sufficient. The same local dimensional reduction was repeated, for the case of 2 × 2 patches, and the distribution of required subspace dimensionality to achieve 98% reconstruction accuracy is shown in Figure 5 (right). As can be seen, for 2 × 2 patches, up to 18-dimensional subspace is required. Thus, it is concluded that local dimensional reduction should be performed on the level of individual pixels, as to maintain the fine details of each pixel’s history while keeping the subspace dimensionality low as possible. We note that the addition of an adaptation rate parameter (the relative importance of the new coming data vector at each incremental step) might slightly alter the distribution shown in Figure 5 (left).

### 3.5. Hyperspectral Background Modeling Based on Local Dimensional Reduction

#### 3.5.1. Motivation

*Background modeling* (or *background subtraction*) and the related foreground detection (or change detection) are important steps in video surveillance and well-established themes in computer vision (for an exhaustive survey, see the book [9]). Conventional background modeling methods exploit the temporal variation of each pixel to model the background. There is little previous literature concerning background modeling for multispectral sensors, and even less so for hyperspectral sensors. Since hyperspectral data have strong correlations between adjacent spectral bands and high redundancy, dimensionality reduction techniques are required. Some previous computer vision approaches have employed subspace learning techniques for background modeling, but in an entirely different way than in this work [9], notably using whole frames as data input. In contrast, over time, our approach builds and updates a background subspace for each pixel individually. 

An additional contribution of our work relates to the application of the background modeling algorithm to the case of fragmented occlusion. Fragmented occlusion is characteristic of the scenarios of FOLDOUT, namely through-foliage detection in the context of border crossings. Fragmented occlusions are much more challenging than ordinary partial occlusions as typically only small fragments of the persons are seen through the foliage. Currently, state-of-the-art person detectors based on deep learning fail to detect persons under considerable fragmented occlusion [4]. The motivation of the present work was to develop a background modeling algorithm specifically to detect moving objects (in particular, moving persons) under fragmented occlusion, using a hyperspectral sensor, due to its extended bandwidth relevant for vegetation detection.

#### 3.5.2. The Background Modeling Algorithm

**Initialization** **:**

The algorithm does not assume a training period, where all variations of the dynamical background can be learned offline. The algorithm is planned to work as soon as the sensor is installed within a surveillance system and requires only a few frames for initialization. The algorithm uses the first $N_{batch}$ recorded frames (typically, $N_{batch}=10$) to perform an initial batch-PCA for each pixel individually. Namely, given a 217 × 409 × 25 demosiaced hyperspectral frame recorded at time *t*, each pixel $I_{k}^{t}$(i,j) is a 25-dimensional vector. For each pixel (i,j), a batch-PCA is performed on the data ${\left\{I_{k}^{t}\left(i,j\right)\right\}}_{t=0,\ldots ,N_{Batch}-1}$. The resulting principal subspace serves as the initialization of the background model for the pixel (i,j). Namely:

(2) $${\left\{u{\left(i,j\right)}_{m}^{t},\lambda {\left(i,j\right)}_{m}^{t}\right\}}_{m=1,\ldots ,\dim\left(i,j\right)}.$$

$u_{m}$, $\lambda_{m}$ and $\dim\left(i,j\right)\;$are the eigenvectors, eigenvalues and the dimension of the principal subspace of the pixel $\left(i,j\right)$, and $\dim\left(i,j\right),\;\mathrm{respectively}$ (we omit the time index t when not necessary). The dimension of the principal subspace is set according to a predefined reconstruction accuracy, acc, (proportion of variance explained), which for PCA is given by [22]

(3) $$\frac{\sum_{m=1}^{\dim\left(i,j\right)}\lambda_{m}}{\sum_{m=1}^{d}\lambda_{m}}\;\geq acc.$$

We have used acc=98%. In fact, since $N_{batch}<d=25$, the PCA can be performed more efficiently on a $N_{batch}\times N_{batch}\;$covariance matrix rather than on the original d×d covariance [22].

The segmentation of a new coming pixel $I^{t}\left(i,j\right)$ into background/foreground is based on the projection of the new coming pixel onto its background subspace and computing the magnitude of the part which is orthogonal to the subspace:

(4) $$I^{t}{\left(i,j\right)}^{⊥}=I^{t}\left(i,j\right)-\sum_{m=1}^{\dim\left(i,j\right)}\left(u_{m}u_{m}^{T}\right)I^{t}\left(i,j\right).$$

$\overset{\dim\left(i,j\right)}{\underset{m=1}{\sum }}\left(u_{m}u_{m}^{T}\right)$ is the projection matrix into the subspace spanned by the principal vectors. The projection error is computed via the $L_{1}$ norm, namely:

(5) $$res^{t}\left(i,j\right)=\|I^{t}{\left(i,j\right)}^{⊥}\|{}_{1}.$$

Additionally, for each pixel, the sliding window median and standard deviation are computed over a short history of each pixel ($N_{win}$ frames). Once the moving median $mov\_med^{t}\left(i,j\right)$ is estimated, the moving standard deviation $mov\_std^{t}\left(i,j\right)$ is computed in a robust manner [26]. First the $mov\_med^{t}\left(i,j\right)$ is re-estimated within the latest $N_{win}$ frames window, including the newest frame:

(6) $$mov\_med^{t}\left(i,j\right)=median\left(res^{t}\left(i,j\right)\right).$$

Next, the Median Absolute Deviation (*MAD*) is re-estimated via:

(7) $$MAD^{t}\left(i,j\right)=median\left(\left|res^{t}\left(i,j\right)-mov\_med^{t}\left(i,j\right)\right|\right)$$

The robust re-estimated standard deviation is given by [9]:

(8) $$mov\_std^{t}\left(i,j\right)=1.4821\cdot MAD^{t}\left(i,j\right).$$

The moving-window-based median and standard deviation of the residuals $res^{t}\left(i,j\right)$ provide statistical measures of the dynamical behavior of the background pixel $\left(i,j\right)$. 

**Segmentation (labeling)**: 

The classification of a pixel $\left(i,j\right)$ into background/foreground is carried out according to the following criterion:

(9) $$Label\;of\;I^{t}\left(i,j\right)=\left\{\begin{array}{cc} Foreground, & \begin{array}{c} res^{t}\left(i,j\right)\geq Thresh\cdot \left(25-dim^{t}\left(i,j\right)\right)\;\wedge \\ \left|res^{t}\left(i,j\right)-mov\_med^{t}\left(i,j\right)\right|\;\geq 3\cdot \;mov\_std^{t}\left(i,j\right) \end{array}\\ Background, & Otherwise \end{array}\right..$$

Typically, Thresh=10, and the factor $\left(25-dim^{t}\left(i,j\right)\right)$ is the dimension of the residual vector perpendicular to the principal subspace $I^{t}{\left(i,j\right)}^{⊥}$. The first condition in Equation (9) for the pixel $\left(i,j\right)$ to be classified as a foreground pixel is a common one in the literature, which is based on a fixed global threshold for all pixels [10]. However, as previously discussed [15,27], such a global approach is not sufficient, as it assumes that all pixels within the frame will always present identical behavior throughout time. Moreover, different areas within the frame show different dynamical behavior; some background pixels might be more unstable than others and show more continuous change over time. The second condition in Equation (9) for the pixel $\left(i,j\right)$ to be classified as a foreground pixel, accordingly, serves as a feedback mechanism, to adjust the labeling threshold based on the history of the pixel $\left(i,j\right)$. In contrast with previous efforts [15,27] which employ ad hoc formulas and additional parameters to achieve a feedback mechanism, our approach uses a moving average-based robust statistics of the residual vectors $res^{t}\left(i,j\right)$ for each pixel $\left(i,j\right)$, to adapt to pixels’ instability over time. For pixels with larger dynamical instability (e.g., due to swaying vegetation and lighting flickering), a larger value of $res^{t}\left(i,j\right)$ is required to be labeled as a foreground pixel, compared to a pixel which is more stable over time. We have found that a moving window of width of 5 frames to works better than longer windows (e.g., 20 frames). This is in accordance with the fact that we do not aim to employ a long training period to learn all possible background modalities of all pixels, which we find to be unrealistic. The result of the segmentation process is a foreground mask.

**Post-processing the foreground mask**: 

Preventing the mislabeling of most (if not all) of the dynamical background pixels as foreground requires additional post-processing steps on the foreground mask. These mislabeled pixels correspond to swaying vegetation and illumination changes, as motion of one layer of vegetation exposes hitherto occluded deeper layers of vegetation. Traditional methods such as morphological operations on foreground blobs, foreground mask filling via dilation-erosion, noise removal via filtering, and final contour detection are appropriate when the detected objects are large enough compared to the noise. In our case, the detected persons could be both small and occluded, such that only small fragments of the persons are visible. Filtering might remove those fragments as well. Therefore, we have only used gentle filtering where isolated foreground pixels were removed, provided that their 5 × 5 neighborhood is empty. 

To deal with dynamical background, spatial awareness is required as well. We have employed optical flow estimation. Denoting the spatial and temporal derivatives by $I{\left(i,j\right)}_{x}$, $I{\left(i,j\right)}_{y}$ and $I{\left(i,j\right)}_{t}$, respectively, the optical flow (velocity) vector at pixel $\left(i,j\right)$ is computed using a small neighborhood N (we have used 3 × 3 or 5 × 5), as follows [28]:

(10) $$\left[\begin{array}{c} u_{k}\left(i,j\right)\\ v_{k}\left(i,j\right) \end{array}\right]={\left[\begin{array}{cc} \overset{}{\underset{\left(x,y\right)\in N}{\sum }}I_{x}{\left(i,j\right)}^{2} & \underset{\left(x,y\right)\in N}{\sum }I_{x}\left(i,j\right)I_{y}\left(i,j\right)\\ \underset{\left(x,y\right)\in N}{\sum }I_{y}\left(i,j\right)I_{x}\left(i,j\right) & \underset{\left(x,y\right)\in N}{\sum }I_{y}{\left(i,j\right)}^{2} \end{array}\right]}^{-1}\left[\begin{array}{c} -\underset{\left(x,y\right)\in N}{\sum }I_{x}\left(i,j\right)I_{t}\left(i,j\right)\\ -\underset{\left(x,y\right)\in N}{\sum }I_{y}\left(i,j\right)I_{t}\left(i,j\right) \end{array}\right]$$

We note that computing the optical flow in the reduced subspace rather than in the full 25-dimensional space would require projecting the entire neighborhood of each pixel to the pixel’s subspace. As this could be computationally demanding, it is left to future work. Instead, the velocity vectors $\left(u_{k}\left(i,j\right),\;v_{k}\left(i,j\right)\right)$ were computed without dimensional reduction, keeping in mind that the purpose of Equation (10) is not to accurately estimate the optical flow, but rather to prune out false positive foreground pixels, based on inconsistency of their motion (and violation of the brightness constancy assumption). Experimentation with Equation (10) in a forest scenario revealed that false positive foreground pixels are distinguished by their very small velocity values. Often the motion of swaying vegetation is rather small. Flickering pixels, which result from the multi-layer structure of the vegetation, where swaying of on layer of vegetation exposes a deeper layer to a sudden illumination change, is characterized by brightness inconsistency and turns out to yield a low velocity value. Accepted foreground pixels are those for which there exists at least one spectral band *k*, for which:

(11) $$\left|u_{k}\left(i,j\right)\right|\geq 0.25\;\wedge \;\left|v_{k}\left(i,j\right)\right|\geq 0.25.$$

The foreground mask $FG^{t}\left(i,j\right)$ is temporally smoothed via

(12) $$FG^{t}\left(i,j\right)\to \;\left(1-\alpha_{FG}\right)\cdot FG^{t-1}\left(i,j\right)+\alpha_{FG}\cdot FG^{t}\left(i,j\right).$$

We have used $\alpha_{FG}=0.25$. Finally, a detection alarm is raised only in the case that sufficient evidence is found in the temporally smoothed foreground mask—namely, that at least 25% of region of size 10 × 5 consists of foreground pixels. The small region size of 10 × 5 is taken so as not to assume any specific person size.

**Updating the background**: 

Given a new coming frame, an incremental PCA for each pixel is performed, and its principal subspace is updated according to Section 3.3.2. The incremental PCA increases the dimension of the subspace by one; however, Equation (3) is used to find the minimal subspace dimension required to maintain the reconstruction accuracy of 98%. In contrast with previous literature, such as [27], the background is updated regardless of the classification of a pixel as background or foreground, as the background model needs to be adapted for all variations of background appearances, including dynamical behavior of pixel’s static. However, in the case that a pixel is labeled as foreground, an update of the background is performed sub-temporally, with a probability of 0.75. The outline of the background modeling algorithm is summarized in Algorithm 1.
**Algorithm 1:** Hyperspectral Background Modeling**Initialization**: • Perform pixel-based batch-PCA on first $N_{batch}$ frames to obtain the principal subspace for each pixel, ${\left\{u{\left(i,j\right)}_{m}^{t},\lambda {\left(i,j\right)}_{m}^{t}\right\}}_{m=1,\ldots ,\dim\left(i,j\right)}$ • Compute moving window median and standard deviation $mov\_median^{t}\left(i,j\right)$, $mov\_std^{t}\left(i,j\right)$ based on $N_{win}$ frames. **for** each 25-dimensional pixel in the new coming frame $I_{k}^{t}\left(i,j\right)$      • Project the pixel to its current principal subspace ${\left\{u{\left(i,j\right)}_{m}^{t},\lambda {\left(i,j\right)}_{m}^{t}\right\}}_{m=1,\ldots ,\dim\left(i,j\right)}$      • Label the pixel as background/foreground according to the residual vector perpendicular to the subspace $res^{t}\left(i,j\right)=I^{t}{\left(i,j\right)}^{⊥}{}_{1}$      • Update $mov\_median^{t}\left(i,j\right)\;$and $mov\_std^{t}\left(i,j\right)$      • Post-process the resulting foreground mask:               ○ Remove pixels with inconsistent (slow) motion               ○ Filter isolated pixels               ○ Smooth the foreground mask temporally               ○ Accumulate evidence on the foreground mask               ○ Raise detection alarm if moving objects detected      • Update principal subspace and its dimensionality ${\left\{u{\left(i,j\right)}_{m}^{t},\lambda {\left(i,j\right)}_{m}^{t}\right\}}_{m=1,\ldots ,\dim\left(i,j\right)}$
**End**

## 4. Results

The purpose of the present work is not to provide a detailed evaluation and comparison of the proposed background modeling algorithm. There is little previous literature concerning background modeling with hyperspectral sensors, not to mention novel background modeling approaches that were developed specifically for a hyperspectral sensor. To the best of our knowledge, there are no hyperspectral datasets, certainly not for the case of fragmented occlusion. Moreover, there are only few previous efforts dedicated to the application of hyperspectral sensors in the area of ground-based surveillance. Our goal is twofold: on one hand, we aim to introduce a novel dedicated hyperspectral background modeling algorithm, and on the other hand, we aim to apply it to ground-based surveillance scenarios of FOLDOUT, namely, through-foliage detection on border crossings, which contain a large amount of fragmented occlusion. We follow this approach as first the hyperspectral sensor has an extended bandwidth, where vegetation shows a higher reflectance, and second as conventional DNN-based detectors perform inadequately in the case of large amount of fragmented occlusion.

An additional difficulty in any evaluation of detection algorithms in the case of heavy fragmented occlusion is the issue of annotations. As discussed in [4], under considerable fragmented occlusion, the detected object appears in the video frame as a collection of a few small patches of pixels. Even for a human annotator, it is not possible to place a reasonable bounding box that circumscribes the targets to be detected. Consequently, the evaluation we have performed is based on labeling of whole frames rather than labeling pixels individually. The frames of the video clip were annotated as occupied by persons or empty. Thus, a positive sample in our evaluation is a frame where some evidence of at least one person appears, even if it is just a small patch comprised of a few pixels. A true positive is a positive sample frame for which at least one region of the foreground mask obtained consists of sufficient foreground pixels.

For the evaluation, a video clip acquired within FOLDOUT was used. The video was recorded at Imatra, Finland, in May 2019, depicting a forest scenario where persons are walking behind trees. The clip was recorded with 15 fps and is about 2 min long. In total, 1675 frames were annotated as described above. The amount of fragmented occlusion varies mostly from moderate to heavy. The scenario is comprised of dynamical background including swaying vegetation—grass, bushes, leaves, and branches—and the corresponding light variations and flickering, as a swaying layer of vegetation exposes a deeper layer further from the camera to a sudden illumination change. A direct comparison of our background-based detector against the state-of-the-art DNN-based detector is not possible, as we do not have a dedicated DNN-based detector for the hyperspectral sensor. (For the difficulties involved, see [5]).

**Figure 6:**
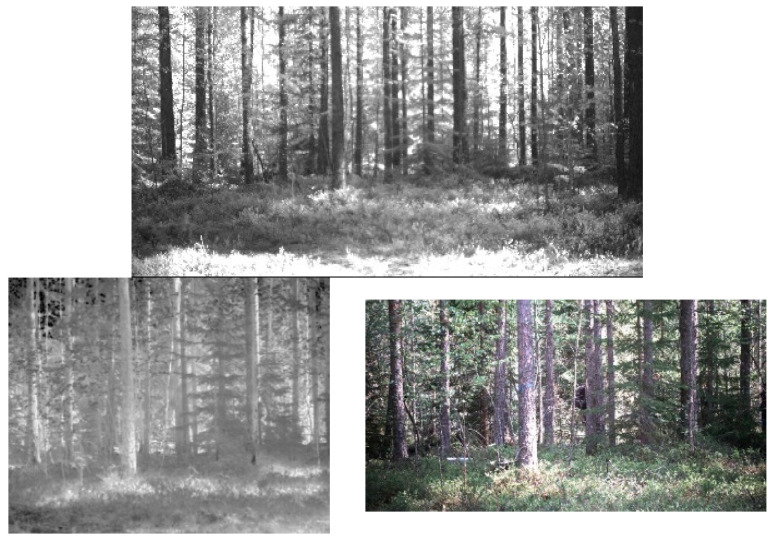
Comparison of the field of view of hyperspectral (**one spectral band**) vs. the thermal (**bottom left**), and RGB (**bottom right**) sensors.

However, the recording of the video clip in Finland was accompanied by simultaneous recordings of the same scenario with RGB and thermal cameras, although with non-identical field of views. Figure 6 shows one frame for each of the three sensors (for the hyperspectral sensor, only one spectral band is shown, for clarity). In FOLDOUT, state-of-the-art DNN-based YOLOv5 detector was implemented for both the RGB and the thermal sensors. An evaluation was performed with the YOLOv5 on the corresponding thermal and RGB recordings, and, likewise, considering a true positive a positive sample frame, where the detector succeeded to detect at least one person in the frame. The YOLOv5 detector managed to detect only 3% of the positive samples of the thermal sensor, and 12% for the RGB sensor, notably, with 0% false positives. The result for the RGB sensor is slightly better compared to the thermal sensor, presumably due to the fact that the field of view is narrower, hence small patches of the occluded persons contain more pixels, as well as due to the fact that some heavy occlusion regions are excluded from the RGB frames. Compared with the low detection rate of DNN-based detectors, the novel hyperspectral background modeling-based detector achieves much higher detection rate; however, it comes at the cost of an increased false alarm rate. With the parametrization of the algorithm, as described in Section 3.5.2, the detection rate is 93%, with a false positives rate of 7%.

**Figure 7:**
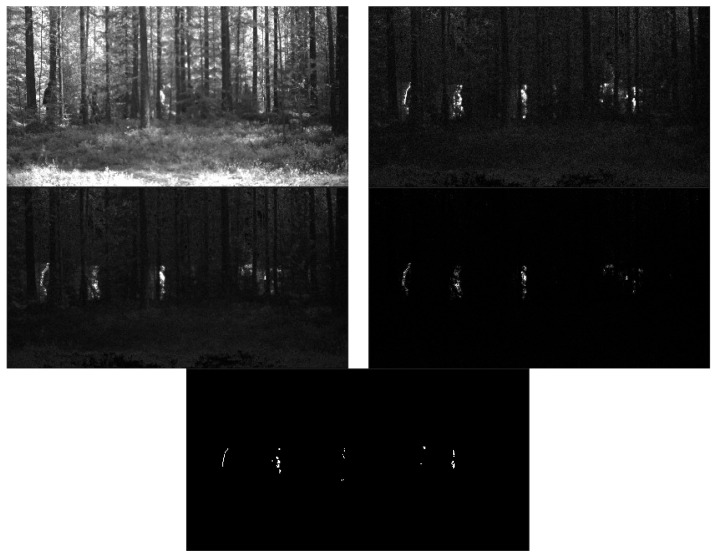
Illustration of the algorithm. **Top left**: original frame (one spectral band). **Top right**: residual ($L_{1}$-norm) image. **Middle left**: moving median. **Middle right**: moving standard deviation. **Bottom**: post-processed foreground mask.

Next, Figure 7 illustrates the background modeling algorithm. The frame 1325 is show (top left), which contains five persons (only spectral band 11 is shown, for clarity). It was chosen for display as it shows persons with various degrees of occlusions, and not only heavily occluded persons, as it is often the case. The top right image is the residual ($L_{1}$-norm) of projecting each new coming pixel to its principal subspace. The middle left and right shows the moving median and moving standard deviation images. The post-processed foreground mask (dynamical background eliminated) is shown at the bottom.

## 5. Discussion

This work presents a novel background modeling (background subtraction) algorithm for a hyperspectral sensor, in the context of ground-based outdoor surveillance. 

The algorithm copes with the strong correlations between adjacent spectral bands and high redundancy by performing an incremental PCA for each pixel individually, maintaining and adapting the principal subspace for each pixel over time. The algorithm was developed within the project FOLDOUT, which focuses on through-foliage detection in border scenarios. Through-foliage scenarios are characterized by heavy fragmented occlusion. On these scenarios, state-of-the-art DNN-based detectors, such as YOLOv5 and Mask-RCNN, perform inadequately [4]. The hyperspectral sensor was investigated in FOLDOUT due to its extended spectral bandwidth, beyond the range of a typical RGB sensors, where vegetation has pronounced reflectance. In outdoor scenarios, the spectral signature obtained from hyperspectral imaging depends significantly on the varying and uncontrolled illumination, which poses a problem for classification methods, where the knowledge of the reflectance of the objects (materials) themselves is required. In contrast, the background modeling approach does not rely on a spectral signature, as it adapts to illumination changes over time. 

Hence, a dedicated background modeling for the hyperspectral sensor was developed and tested on through-foliage penetration scenarios. To the best of our knowledge, there are very few previous studies which focus on ground-based surveillance using a hyperspectral camera, and on developing novel background modeling algorithms for the hyperspectral sensor. Moreover, we demonstrate the algorithm on a challenging through-foliage penetration scenario. Such a scenario is characterized by heavy fragmented occlusion, where only small fragments of the persons walking behind vegetation can be seen, and where the background is highly dynamical, including frequent swaying vegetation and illumination changes. Furthermore, the observed fragments of the moving persons are often comprised of only a few pixels. 

The present work was intended as a demonstration of ground-based outdoor surveillance using a hyperspectral camera, for the purpose of through-foliage detection, employing a dedicated novel background modeling algorithm, rather than a detailed evaluation of the proposed algorithm. Moreover, to the best of our knowledge, there are no hyperspectral datasets or approaches to compare, certainly for through-foliage detection. Our method is demonstrated by evaluation on a video clip acquired within FOLDOUT, with settings reminiscent of border crossing region. The clip contains about 2 min (about 2000 frames) of persons under considerable fragmented occlusion walking behind trees. The video was manually annotated, where whole frames were classified as occupied with persons (positive samples) or devoid of persons (negative sample). A frame was classified as occupied when at least one small fragment of one person was visible. Due to the heavy fragmented occlusion, it was not feasible to annotate persons via their bounding boxes. As no DNN-based classifier was developed for the hyperspectral sensor (for the difficulties involved, see [5]), we compare the performance of our background modeling algorithm to state-of-the-art DNN-based YOLOv5 detector indirectly. As thermal and RGB videos were recorded simultaneously with the hyperspectral video on the same scenario, we ran the YOLOv5 detector on the corresponding thermal and RGB videos. The comparison is shown in the following Table 1.

A true positive frame designates the case where at least one object was detected. As can clearly be seen, the DNN-based detector performs poorly in the case of considerable fragmented occlusion. This was further discussed in [4], which shows that even when the training of a DNN-detector is augmented with fragmented occlusion, the performance in the case of moderate-to-heavy fragmented occlusion does not improve. In contrast, the background modeling-based detectors cope successfully with the moving persons, even when only small fragments of the persons are visible. The success of the background modeling algorithm comes with a trade-off, nevertheless: namely 7% false positive rates, due to the dynamical nature of the background on one hand, and the small visual extent of the moving targets on the other hand. Reducing the false positive rate might be achieved via improvement in the hyperspectral technology, by the planned future work on the background modeling algorithm, as well as from fusion with other algorithms and other sensors. However, the proposed approach demonstrates the feasibility of incorporating a hyperspectral sensor in computer-vision-based surveillance, and the through-foliage penetration scenario is one in which the hyperspectral sensor might excel.

Since the purchase of the XISPEC-KIT-SM5X5-NIR (Generation 1; XIMEA GmbH, Münster, Germany)hyperspectral sensor within FOLDOUT during 2019, there has been improvement in hyperspectral sensor technology. Additionally, with additional future advancement, it is foreseen that hyperspectral sensors with reduced correlations between adjacent spectral bands, redundancy, and noise, and with a reduced price point, will be used more commonly in ground-based surveillance. In particular, with increased resolution, larger fragments of moving targets should be easier to detect and classify as such, and texture features such as histograms of local binary pattern [14] might be employed.

Our approach maintains and updates a principal subspace for each pixel, thus reducing the number of hyperspectral bands for each pixel by more than 50%. Currently, the algorithm is implemented in MATLAB. However, preliminary tests indicate that real-time performance should be feasible. This could be achieved by using, e.g., Python in combination with TensorFlow to eliminate the computational bottleneck of updating the principal subspace for each pixel separately using parallelization. 

## 6. Conclusions

The main limitation of our approach is the tradeoff between detection rate and false alarms rate. This could be improved by elaborating the structure of our background model. In [12], an efficient representation of a non-parametric description of the background model is outlined using a codebook model. In [29], a simplified version of codebooks was demonstrated to work in ultra-real time in practical outdoor surveillance scenarios, without the need for an offline training period. In [20], an extension of the method for multispectral frames is presented (6–7 spectral bands). In future work, our plan is to augment our hyperspectral background model with additional structure, along these lines, to reduce the number of false positives. For each pixel, we plan to maintain and update a list of codebooks within its principal subspace. Although the FOLDOUT project demonstrated that fusion of various sensors of different modalities improves the detection and tracking of targets [2], the hyperspectral sensor was not yet part of that fusion. This is left for future work.

## Figures and Tables

**Table 1:** Comparison of the background modeling algorithm with dep learning approaches. (TP = true positives; FN = false negatives; FP = false positives; TN = true negatives).

	Background Modeling (Hyperspectral)	YOLOv5 (Thermal)	YOLOv5 (RGB)
True Positive Rate = TP/(TP + FN)	93%	3%	12%
False Positive rates = FP/(TN + FP)	7%	0%	0%

## Data Availability

Not applicable.
